# Amine-Grafted Mesoporous Carbons as Benzocaine-Delivery Platforms

**DOI:** 10.3390/ma14092188

**Published:** 2021-04-24

**Authors:** Joanna Goscianska, Aleksander Ejsmont, Anita Kubiak, Dominika Ludowicz, Anna Stasiłowicz, Judyta Cielecka-Piontek

**Affiliations:** 1 Department of Chemical Technology, Faculty of Chemistry, Adam Mickiewicz University in Poznań, Uniwersytetu Poznańskiego 8, 61-614 Poznań, Poland; aleejs@amu.edu.pl (A.E.); anitakubiak9@gmail.com (A.K.); 2 Department of Pharmacognosy, Faculty of Pharmacy, Poznań University of Medical Sciences, Święcickiego 4, 61-781 Poznań, Poland; dsiakowska@ump.edu.pl (D.L.); astasilowicz@ump.edu.pl (A.S.)

**Keywords:** carbon vehicles, drug delivery, benzocaine, release studies, sorption capacity, kinetics models

## Abstract

Smart porous carriers with defined structure and physicochemical properties are required for releasing the therapeutic drug with precise control of delivery time and location in the body. Due to their non-toxicity, ordered structure, and chemical and thermal stability, mesoporous carbons can be considered modern carriers for active pharmaceutical ingredients whose effectiveness needs frequent dosing algorithms. Here, the novel benzocaine delivery systems based on ordered mesoporous carbons of the cubic structure were obtained with the use of a hard template method and functionalization with amine groups at 40 °C for 8 h. It has been shown that amine grafting strongly modifies the surface chemistry and textural parameters of carbons. All samples indicated good sorption ability towards benzocaine, with evident improvement following the functionalization with the amine groups. The sorption capacity and drug release kinetics were strongly affected by the porosity of carbon carriers and the surface functional groups. The smallest amount of benzocaine (~12%) was released from pristine mesoporous carbon, which could be correlated with strong API–carrier interactions. Faster and more efficient release of the drug was observed in the case of triethylenetetramine modified carbon (~62%). All benzocaine delivery platforms based on amine-grafted mesoporous carbons revealed high permeability through the artificial membrane.

## 1. Introduction

At a time when versatile materials are sought to perform specific functions via appropriate modulations, ordered mesoporous carbons (OMCs) seem to be an endless source of possibilities [1,2]. Their highly tunable properties, such as porosity in the range of 2–50 nm, well-developed surface area, and high thermal and chemical stability, are the base, on top of which further functionalities can be built. Up to now, OMCs and their adsorption capabilities have been applied in various fields, such as hydrogen [3] and energy [4] storage, catalysis [5,6], gas capture [7,8], water treatment [9,10], and drug delivery [11,12]. Whilst the latter application of OMCs as carriers for active pharmaceutical ingredients (APIs) is newly emerging, they are more often considered due to their confirmed biocompatibility [13,14,15]. Ordered carbons obtained by the hard-template method, where silica is used as a matrix and the carbon source is highly purified, can be stable, non-toxic carriers without contamination and heavy metal residue [16,17,18]. The use of carbons as drug carriers is intended to improve the delivery of APIs characterized by poor solubility, chemical lability, or/and instability, to the target place in the body [19,20]. Such an API–carrier system if programmed precisely may stabilize the pharmaceutical and increase its dissolution, leading to enhanced bioavailability. The substance conjugated with the material, through its prior adsorption, can later be successfully applied internally to the organism, as well as externally if the API release is feasible under these conditions.

In our study, we synthesized OMCs based on the KIT-6 silica (KIT—Korea Advanced Institute of Science and Technology) via nanocasting method to receive material with good sorption abilities. Subsequently, the carbon was functionalized with different amines to increase its affinity to benzocaine, and also to allow the material to liberate it in an environment similar to the oral cavity. All of it was determined by the sorption and release studies, along with the OMCs physicochemical characterization. The chosen API was benzocaine, known for its local anesthetic effect, which occurs through inhibiting the voltage-dependent sodium channels [21]. The action mechanism is based on blocking the propagation of an action potential, which means that the brain does not receive an impulse that would be identified as pain. Although benzocaine has been known as a local pain reliever for quite some time—its synthesis was carried out for the first time at the end of the 19th century—it is still used in medicine. This is mainly due to its relative safety—the most common side effects while applying topically are hypersensitivity reactions; serious ones, such as methemoglobinemia, are rare [22]. It is classified as a compound belonging to Class II of the Biopharmaceutical Classification System due to its high permeability and low water solubility. The onset of benzocaine action can be observed less than 1 min after application, and the duration of action is about 5–10 min. Taking into account the use of benzocaine in formulations acting within the oral cavity, e.g., in case of sore throat, ulcers, injuries resulting from the use of prostheses or dental braces, and inflammations of the pharynx or larynx, the prolonged anesthetic effect would be beneficial, as it would provide more lasting relief in the indicated ailments. So far, there have been proposals to use liposomal systems as carriers for other local anaesthetic substances. One of the registered examples is EXPAREL (Pacira BioSciences, Inc., San Diego, CA, USA), which is a liposomal form of bupivacaine [23]. Dependently on the place of pain, benzocaine usually is applied topically, e.g., in an ointment or gel. However, most often it is administrated through the oral/oromucosal route, either in the form of spray or lozenges. Up to now, it was proposed to load benzocaine into polymerized vinylbenzaldehyde [24] or solid-lipid nanoparticles [25], with no evident carbonaceous carriers in use. For instance, proniosomal gels were proposed as semisolid non-ionic surfactant-based (i.a. Span 60) carriers for benzocaine [26]. Whilst such systems revealed sustained profiles of anesthesia duration, their safety can be concerning. In early reports, it was stated that Span 60 does not exhibit long-term toxicity, nor carcinogenicity; however, a significant incidence of nephrosis in mice was noted [27]. Other materials with confirmed good biocompatibility, e.g., lactide- or caprolactone-based polymers in a form of polymeric nanocapsules with entrapped benzocaine have been developed [28,29]. The colloidal system provided high encapsulation efficiency and drug loading, up to 73.3% and 6.67% (*w*/*w*), respectively. Moreover, it is typical that between nanocapsules and API occurs strong affinity; therefore, to liberate pharmaceutics, the degradation of the carrier is necessary. In the respect of mentioned benzocaine carriers, porous carbons come up as new generation materials with good bioavailability, along with the efficient and less troublesome release. Weaker interactions in the OMC-API system enable modulating benzocaine release from gradual to rapid depending on carbon functionalities. Up to now, the only record of the interaction of benzocaine with porous carbon material was its adsorption onto activated carbon (AC) in 1990 [30]. Microporous ACs with irregular and very small pores are not suitable for precise API carrier development where pore uniformity and ordering are significant to predict adsorption-release behavior. Moreover, ACs usually exhibit hydrophobicity and strong adsorption. When, due to the first trait, poor wettability impeded API dispersion and its release, as, e.g., in Miriyala et al.’s work [31], to increase paracetamol and ibuprofen liberation, a surfactant (SDS) was added. The high sorption capabilities of ACs seem to give them an upper hand over other drug vehicles [32]. In fact, the adsorption in micropores is either too strong to release all adsorbed API or pores are too small (<2 nm) to adsorb drug molecules, which usually leads to non-controlled release. Mesoporous carbons are able to efficiently adsorb but also to release API, if only appropriate modifications are conducted. In our case, oxidation was used to create surface functionalities utilized as anchors to attach amines. In other research on drug delivery, amines were applied to functionalize the surface of silica carriers, e.g., MCM-41 (MCM—Mobil Composition of Matter) and SBA-15 (SBA—Santa Barbara Amorphous) for ibuprofen [33,34]. Whilst silica nanoparticles are broadly used as API vehicles, their toxicity especially on the immune system has been unsatisfactorily revised. For instance, silica nanoparticles can be responsible for the change of activity and behavior of macrophages/monocytes [35]. Therefore, the search for different types of materials less toxic and biocompatible is necessary. 

Our goal was to receive carbon materials with enhanced sorption abilities towards benzocaine and increased release due to the amines functionalization. Additionally, we aimed to achieve controlled release of benzocaine from the carbon carriers, in order to prolong the action of the drug, and in perspective to reduce the frequency of its use. Benzocaine is sparingly soluble in water, and taking it in large doses in the oral cavity does not increase its action but can only cause it to pass deeper into the digestive system. As a consequence, the drug is not dissolved in the desired place and the patient will not receive the absolute therapeutic effect and full pain relief. In the end, it leads to the need to take the medicine again. However, the use of a carrier may contribute to the reduction of the dosage as well as the lowering of the benzocaine concentration in the drug formulation, e.g., in lozenges.

## 2. Experimental

### 2.1. Materials

Pluronic P123 (BASF, Ludwigshafen, Germany), hydrochloric acid (35–38%, Chempur, Piekary Śląskie, Poland), 1-butanol (POCH, Gliwice, Poland), tetraethyl orthosilicate (TEOS, 98% wt., Aldrich, Poznan, Poland), sucrose (99.5% wt., Aldrich), sulphuric acid (Chempur, 98%), hydrofluoric acid (40%, Aldrich), ammonium persulfate (98%, Aldrich), ethanol (Chempur), ethylenediamine (EDA, Aldrich), diethylenetriamine (DTA, Aldrich), triethylenetetramine (TTA, Aldrich), tetraethylenepentamine (TPA, Aldrich), methanol (Chempur).

### 2.2. Synthesis of Amine-Grafted Ordered Mesoporous Carbons

The first step was to synthesize KIT-6 silica serving as a hard template. Pluronic P123 (4 g) and hydrochloric acid (7.9 g) were added to a bottle with distilled water (144 mL). The intensively stirred solution was kept in a water bath at 35 °C for 2 h, then 1-butanol (4 g) was poured in and mixed for 1 h. After polymer dissolution, TEOS (8.6 g) was instilled dropwise. Thereafter, the solution with continuous stirring was left for the first 24 h at 35 °C, and for another 24 h at 100 °C. The received precipitate was separated without cooling and dried in the oven for 24 h at 100 °C, afterwards it was calcined for 8 h at 550 °C.

Carbon material C_KIT-6_ was prepared via the nanocasting method. Sucrose (1.4 g) and sulphuric acid (0.14 mL) were dissolved in water (6 mL), to which KIT-6 (1 g) was added. Everything was heated for the first 6 h at 100 °C and the second 6 h at 160 °C. The material was later treated with the impregnation aqueous solution (5 mL) of sucrose (0.8 g) and sulphuric acid (0.09 mL). Subsequently, everything was heated again in the same way—for 6 h at 100 °C and 6 h at 160 °C. The received composite was carbonized with the heating rate of 5 °C min^−1^ for 8 h at 900 °C (argon atmosphere). Carbonized material was subjected to a hydrofluoric acid solution (5 wt.%) at room temperature to remove the silica template; in the end, it was filtrated, washed, dried for 4 h at 120 °C and ground.

To generate oxygen functional groups on the carbon surface, C_KIT-6_ (16 g) was treated with oxidizing solution (960 mL) of ammonium persulfate (1 M) in sulphuric acid (2 M) at 60 °C for 6 h. Washed and dried at 100 °C material was divided into five portions, four of which were functionalized with amines. EDA, DTA, TTA, and TPA (1.6 g were dissolved separately in methanol (30 mL) for 30 min at 40 °C with continuous stirring. Afterward, to each solution, C_KIT-6_ (4 g) was added and mixed for 8 h. In the end, all carbons were filtered, washed with methanol, and dried in the oven overnight at 100 °C. Samples were designated according to the corresponding amine functionalization as C_KIT-6_−EDA, C_KIT-6_−DTA, C_KIT-6_−TTA, and C_KIT-6_−TPA.

### 2.3. Characterization of Materials

#### 2.3.1. Low-Temperature Nitrogen Sorption

The textural parameters of the obtained samples were determined by low-temperature nitrogen adsorption/desorption isotherms. The analysis was conducted at −196 °C with the use of a Quantachrome Autosorb IQ apparatus (Boynton Beach, FL, USA). Prior to measuring, the pristine carbon sample was heated in a vacuum at 300 °C for 3 h, in order to remove the remaining water within the pores. The amine-grafted carbon samples were outgassed at 150 °C for 3 h. The calculations based on the Brunauer–Emmett–Teller (BET) method were used to quantify the surface areas (*S_BET_*) of carbon carriers. The average pore size was evaluated from the adsorption branch of the isotherm by means of the Barret–Joyner–Halenda (BJH) method.

#### 2.3.2. Powder X-ray Diffraction

Mesoporous structure of the carriers in terms of the type and ordering were identified by powder X-ray diffraction. Diffractograms were made at room temperature with a step size of 0.02° in the small-angle range using a D8 Advance Diffractometer (Bruker, Billerica, MA, USA) with the copper Kα1 radiation (λ = 1.5406 Å).

#### 2.3.3. Elemental Analysis

Thermo Scientific FLASH 2000 Elemental Analyzer (OEA, Thermo Fisher Scientific, Waltham, MA, USA) was employed to establish the CHNS/O elemental composition of carbon carriers. 

#### 2.3.4. Infrared Spectroscopy

Fourier transforms infrared spectra were registered with the use of an FT-IR Bruker IFS 66v/S spectrometer (Bruker Optics, Ettlingen, Germany). The carbon samples were studied in the form of pellets obtained by pressing a mixture of anhydrous KBr (ca. 0.25 g) and 0.3 mg of the nanomaterial. The analysis was carried out in a wavenumber range of 4000–400 cm^−1^ (at a resolution of 0.5 cm^−1^; the number of scans: 64).

### 2.4. Adsorption of Benzocaine

The sorption capacity of all samples towards benzocaine was established with the use of an Agilent Cary 60 UV-Vis spectrophotometer (*λ* = 292 nm) (Agilent Technologies, Santa Clara, CA, USA). Each powdered carbon sample (0.05 g) was immersed in benzocaine solutions (50 mL; ethanol: water, volume ratio 1:4) at various concentrations (1–200 mg L^−1^) and agitated on the shaker for 24 h at room temperature. Subsequently, materials were separated from the solutions and thereupon their absorbance was measured. The process was repeated and the standard deviation was calculated from three measurements for each material. The amount of adsorbed benzocaine on the carbons (*q_e_*) was calculated according to the following Equation (1):

(1) $$q_{e}=\frac{\left(C_{0}-C_{e}\right)\cdot V}{m}$$

where:

*C*_0_—initial benzocaine solution concentration (mg L^−1^);
*C_e_*—the benzocaine concentration (mg L^−1^) remaining after the adsorption process;
*V*—the volume of the API solution (L);
*m*—the mass of the carbon vehicle (g).

### 2.5. The Release Studies of Benzocaine

The release study was carried out with the use of dissolution paddle apparatus—Agilent 708-DS complied with USP requirements (Santa Clara, CA, USA). The studied medium (phosphate buffer, pH 6.8) was heated to 37 °C, with a rotation speed of 50 rpm. The adsorption of benzocaine onto carbon carriers was conducted via the evaporation technique. Benzocaine (5 mg, Sigma-Aldrich, Poznan, Poland) was dissolved in 3 mL of ethanol–water solution (volume ratio 1:4), then 25 mg of carbon material was added. The mixture was agitated for 24 h and the solution was evaporated in the oven overnight at 60 °C. To prevent flotation on the surface of the medium, the samples of pure benzocaine and benzocaine introduced into carbon vehicles were placed in the gelatin capsules. At the beginning, the drug release profiles from gelatin capsules and mesoporous carbon systems were compared. Twelve 5 mL samples were withdrawn from each vessel at selected time points. Later, an equal amount of the medium at 37 °C was placed in the vessels. The collected samples were filtered through syringe filters with a pore diameter of 0.45 µm, and their concentration was measured by a high-performance liquid chromatography (HPLC,) method with a DAD detector (Prominence-i LC-2030C, Shimadzu, Kioto, Japan). The stationary phase was a Kinetex-C18 column (100 mm × 2.1 mm, 5 µm, Phenomenex, Warsaw, Poland), and the mobile phase consisted of 0.1% formic acid and acetonitrile (70:30). The injection volumes were 5 µl and the wavelength was set at 292 nm. The process was repeated and the standard deviation was calculated from six measurements for all carbon vehicles.

The benzocaine release from each carbon was compared to five kinetic models via data curve fitting. Zero-order (Equation (2)), first-order (Equation (3)), Higuchi (Equation (4)), Hixson–Crowell (Equation (5)), and Korsmeyer–Peppas (Equation (6)) were employed to determine the correlation coefficient (*R*^2^) and release behavior.

(2) $$F_{t}=k_{0}t$$

(3) $$F_{t}=1-e^{-kt}$$

(4) $$F_{t}=k_{H}\sqrt{t}$$

(5) $$\sqrt[{3}]{F_{0}}-\sqrt[{3}]{F_{t}}=k_{HC}t$$

(6) $$F_{t}=kt^{n}$$

In the formulas mentioned, the meaning of symbols is as follows:

*F_t_*—the fraction of benzocaine released in time;
*F*_0_—the starting amount of benzocaine in the carbon carrier;
*k*_0_, *k*_1_, *k_H_*, *k_HC_*—the release constants of particular kinetic models;
*n*—the diffusion exponent.

The difference (*f*_1_) and similarity (*f*_2_) factors were calculated for the dissolution rate curves according to the Equations (7) and (8):

(7) $$f_{1}=\frac{\sum_{j=1}^{n}\left|R_{j}-T_{j}\right|}{\sum_{j=1}^{n}R_{j}}\times 100$$

(8) $$f_{2}=50\times \log\left({\left(1+\left(\frac{1}{n}\right)\overset{n}{\underset{j=1}{\sum }}{\left|R_{j}-T_{j}\right|}^{2}\right)}^{-\frac{1}{2}}\times 100\right)$$

where *n* is the number of time points, *R_j_* is the percentage of the reference dissolved product in the medium, *T_t_* is the percentage of the dissolved tested product, and *t* is the time point. Based on two-factor values, it is possible to determine the similarity (*f*_1_ in the range of 0–15 and *f*_2_ 50–100) or difference (*f*_1_ above 15 and *f*_2_ below 50) of dissolution profiles [36].

### 2.6. Permeability Studies

The permeability of benzocaine and its systems with mesoporous carbons based on the passive diffusion process was determined with the use of the PAMPA (Parallel Artificial Membrane Permeability Assay) system (Pion, Inc., Billerica, MA, USA). Before testing, samples were prepared in the following way. Benzocaine (5 mg) was dissolved in 3 mL of ethanol–water solution (volume ratio 1:4), then 25 mg of carbon carriers was added. The mixtures were shaken for 24 h and the solutions were evaporated in the oven overnight at 60 °C. The PAMPA system of 96-well plates is divided into two parts: donor and acceptor, separated by microfilter disc. The donor wells were filled with samples, while acceptor wells were filled with acceptor buffer, and the membrane was coated with dodecane solution of a lecithin mixture. Then, both parts of the plate were joined together, and the plate was incubated at a temperature of 37 °C for 3 h. During this time, benzocaine was transported from donor to acceptor through the membrane. When the time passed, the plate was split, and the concentrations of acceptor and donor solutions were measured using a UV spectrophotometer (Multiscan GO, Thermo Fisher Scientific, Vantaa, Finland) set at the wavelength of 292 nm.

The apparent permeability coefficient (*P_app_*) was calculated according to the Equations (9) and (10):

(9) $$P_{app}=\frac{-ln\left(1-\frac{C_{A}}{C_{equilibrium}}\right)}{S\times \left(\frac{1}{V_{D}}+\frac{1}{V_{A}}\right)\times t}$$

(10) $$C_{equilibrium}=\frac{C_{D}\times V_{D}+C_{A}\times V_{A}}{V_{D}+V_{A}}$$

where:

*V_D_*—donor volume;
*V_A_*—acceptor volume;
*C_equilibrium_*—equilibrium concentration;
*S*—membrane area;
*t*—incubation time (in seconds).

According to the literature, compounds with *P_app_* < 0.1 × 10^−6^ cm/s are defined as ones with low permeability, and with a *P_app_* ≥ 1 × 10^−6^ cm/s are referred to as ones with high permeability [37].

## 3. Results and Discussion

The following description of physicochemical properties of amine-functionalized carbons is essential to determine carrier affinity to the selected API. Structure ordering is especially significant because the carbon carriers require to be reproducible and predictable platforms for pharmaceutics.

**Figure 1:**
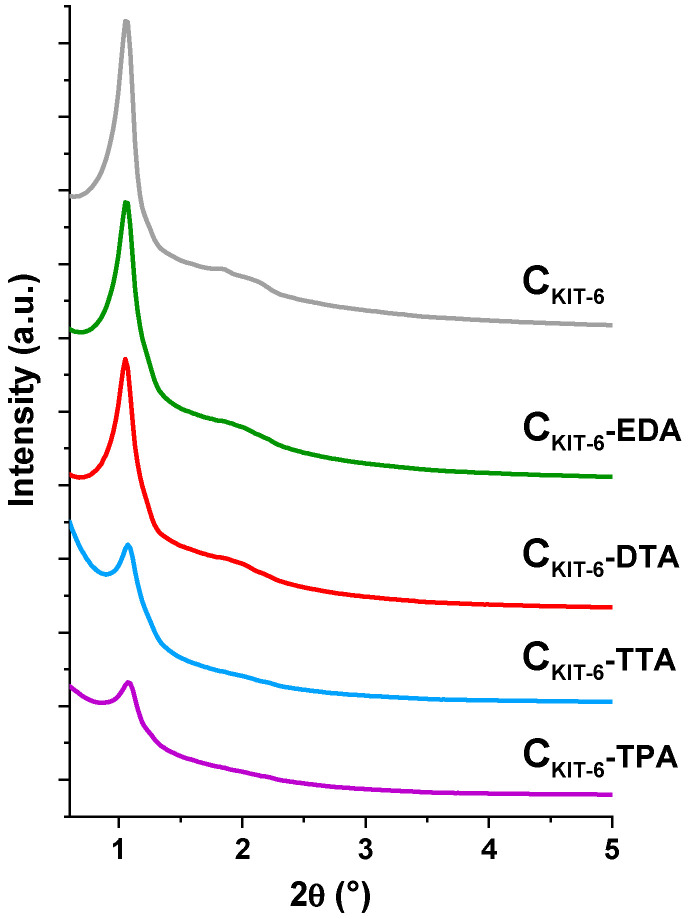
X-ray diffraction patterns in the small-angle range of pristine and amine-grafted ordered mesoporous carbons (OMCs).

It can be stated that pristine mesoporous carbon was successfully synthesized with the use of a KIT-6 silica template. XRD studies conducted in the small 2θ range (0.6–5.0°) confirmed the body-centered cubic Ia3d-type structures of C_KIT-6_ and amine-grafted samples (C_KIT-6_−EDA, C_KIT-6_−DTA, C_KIT-6_−TTA, C_KIT-6_−TPA), as evidenced by the presence of sharp well-resolved (211) reflection at 2θ ≈ 1° and several higher-order peaks at 2θ angles below 3° (Figure 1). The intensity of all reflections decreases with increasing the length of the carbon chain of amines used for the functionalization of pristine carbon. Therefore, the most intense diffraction peaks were observed for non-functionalized carbon C_KIT-6_, and the least sharp peaks for the sample modified with tetraethylenepentamine—C_KIT-6_–TPA. It can be concluded that the functionalization of carbon with amine groups reduces the ordering of cubic mesostructure.

Textural parameters determined by the low-temperature N_2_ adsorption revealed an almost linear dependence of the BET surface area, total pore volume, and average pore diameter to the length of the amines’ carbon chain (Table 1). The specific surface area (834 m^2^ g^−1^) and total pore volume (1.09 cm^3^ g^−1^) are the highest for the pristine material C_KIT-6_. Contrastingly, these values decrease successively, to 189 m^2^ g^−1^ and 0.29 cm^3^ g^−1^, for C_KIT-6_–TPA, respectively. Such a reduction is an effect of a partial micropore/small mesopore-blocking with the grafted amine groups or their damage during the functionalization process. In consequence, the average pore diameters of materials also increase from 5.6 to 6.8 nm with the amines’ carbon chain elongation.

**Figure 2:**
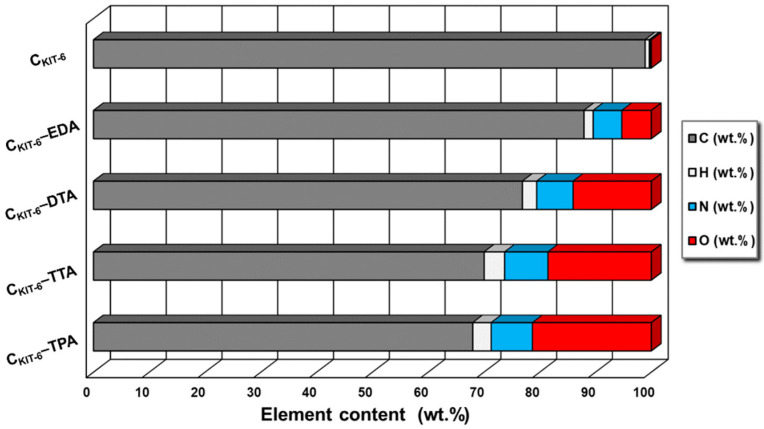
Elemental analysis of mesoporous carbon vehicles obtained.

To prove the presence of nitrogen-containing functional groups on the surface of the carbon materials, the elemental analysis was performed (Figure 2). As expected, the non-modified sample C_KIT-6_ contains a small amount of nitrogen (< 0.5%). Furthermore, it was noted that modification with the use of ethylenediamine, diethylenetriamine, triethylenetetramine, and tetraethylenepentamine leads to an increasing content of nitrogen to 5% for C_KIT-6_–EDA, 6.5% for C_KIT-6_–DTA, 7.8% for C_KIT-6_–TTA, and 8% for C_KIT-6_–TPA. It affirms that a higher number of amine groups in organic compounds allows for the introduction of more nitrogen into the mesoporous carbon carriers.

**Figure 3:**
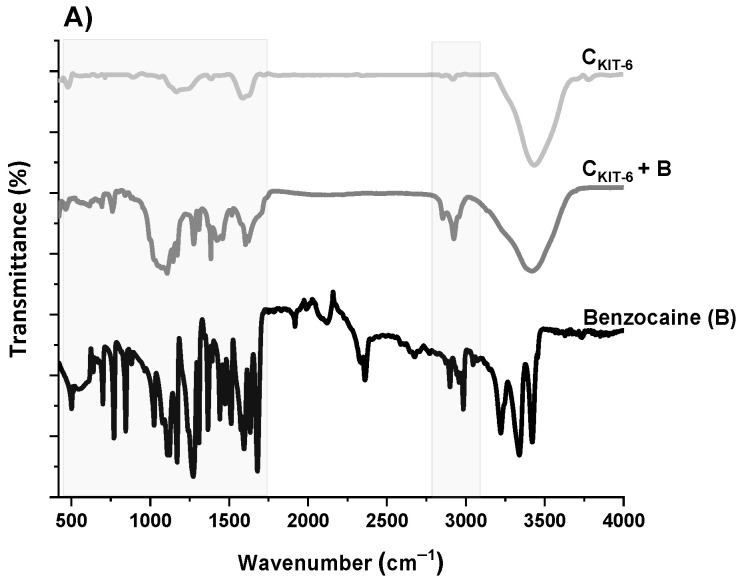

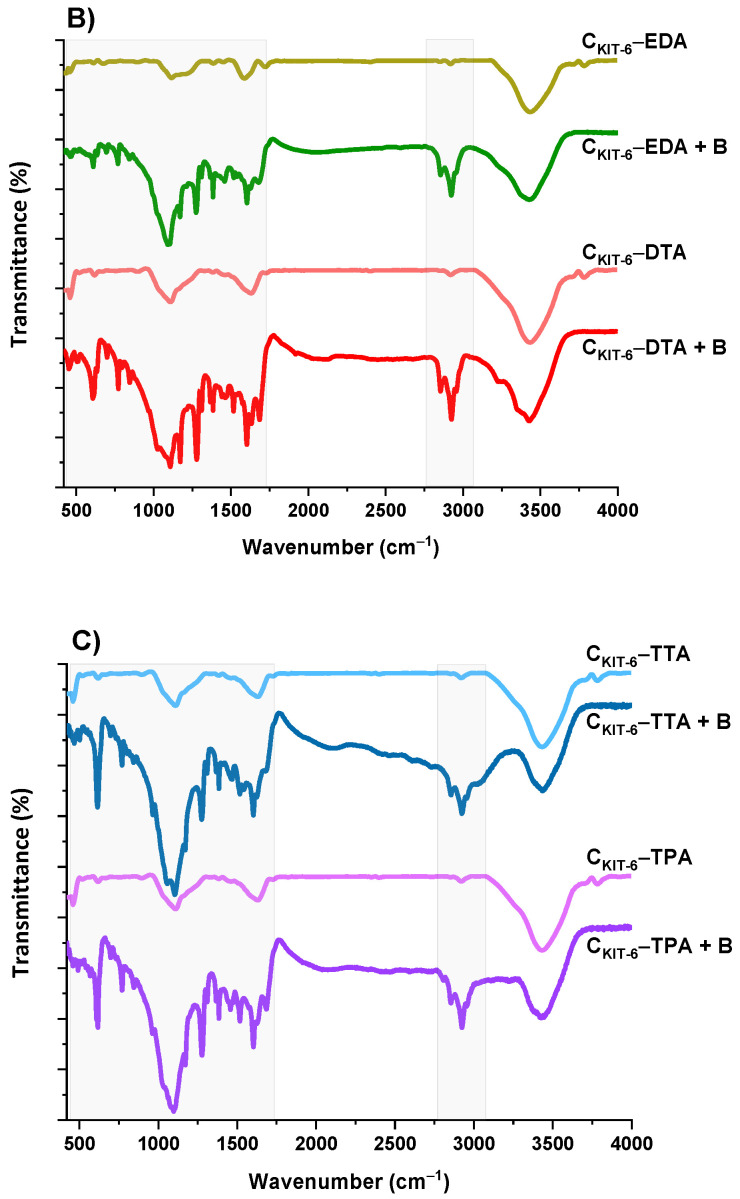
FT-IR spectra of benzocaine (black) and ordered mesoporous carbons before and after adsorption: C_KIT-6_ (gray)—(**A**); C_KIT-6_–EDA (green) and C_KIT-6_–DTA (red)—(**B**); C_KIT-6_–TTA (blue) and C_KIT-6_–TPA (purple)—(**C**).

The next step of the physicochemical characterization of carbon carriers was FT-IR spectral analysis. The FT-IR spectra of all carbon samples, presented in Figure 3, contain similar bands, differing in intensity. The bands at around ~1119 and ~1180 cm^−1^ can be identified as C–O bonds in carboxylic, etheric, phenolic, and ester groups [38]. The band close to 1588 cm^−1^ is characteristic for C=O conjugated in the graphene layer (quinone structure), and also for C=C, along with C–H in the surface aromatic structure [39]. Additionally, the bands at 2918–2950 cm^−1^ and 2844–2870 cm^−1^ prove the presence of symmetric and antisymmetric stretching vibration of the C–H bond, respectively. The broadest band appearing in the range of 3200–3700 cm^−1^ is the most frequent and apparent in various carbon materials, assigned to O–H stretching vibrations that originated from –COOH as well as from the adsorbed water molecules. After the functionalization of the C_KIT-6_ sample with amine groups, in the FT-IR spectra, new bands at 1242, 1161 and 616 cm^−1^ are observed related to C–N stretching, N–H bending and wagging vibrations, respectively, whose intensity varies depending on the complexity of the grafted functionalities [40,41].

**Figure 4:**
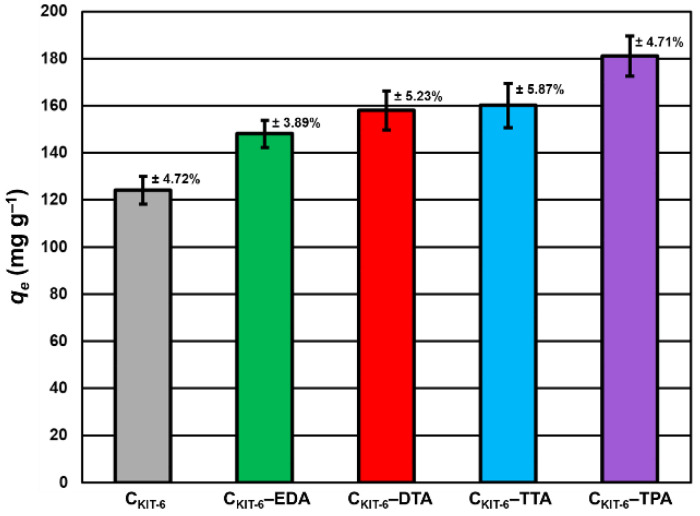
Maximum sorption capacity (*q_e_*) of carbon carriers towards benzocaine. The standard deviation calculated from three measurements for each material has been presented.

To understand how benzocaine binds to the carbon carrier surface, it is also necessary to analyze its FT-IR spectrum (Figure 3A). The characteristic bands in the range of ~450–1750 cm^–1^ and ~2770–3080 cm^–1^ were primarily considered. At ~850 cm^–1^, the wagging vibrations of C–H bonds present in the benzene ring were identified [42]. Contrastingly, at 1102 cm^–1^, the stretching vibrations of C–C bonds of the ring itself are noticed, which correspond to the breathing mode. Non-aryl bands are detected between ~1260 and 1320 cm^–1^, which belong to the stretching vibrations of the C–O and C–C bonds. Moreover, the significant stretching vibration of the C=O bond is noticeable at 1696 cm^–1^. Furthermore, the bands centered at ~2895 and 2985 cm^–1^ assigned to the symmetric stretching vibrations of C–H from –CH_3_ and the asymmetric stretching vibrations of C–H from –CH_2_ are clearly visible. The pronounced three bands in the benzocaine FT-IR spectra at 3218, 3347, and 3427 cm^–1^ can be attributed to the symmetric and asymmetric stretching vibrations of N–H [42]. In the FT-IR spectra of all mesoporous carbon samples registered after the adsorption of benzocaine, some new bands derived from the active substance become visible, especially in the wavenumber range of ~450–1750 cm^–1^. They are definitely more intense in the case of amine-grafted carriers, which indicates their greater efficiency in benzocaine binding compared to pristine carbon C_KIT-6_. Moreover, after benzocaine adsorption, modest shifts in the maxima of certain bands were perceived, suggesting that API is linked to the carbon carriers mostly via weak interactions including hydrogen bonds, van der Waals forces, and electrostatic interactions. The sorption capacities of the pristine and amine-grafted mesoporous carbon carriers with respect to benzocaine are depicted in Figure 4. All samples revealed good sorption ability, with evident improvement following the functionalization with the amine groups. Thanks to a high specific surface area (834 m^2^ g^−1^) and pore volume (1.09 cm^3^ g^−1^), pristine ordered mesoporous carbon by itself has accessible active sites which allow for the effective binding of adsorbate molecules [43]. Thus, the C_KIT-6_ sample even without grafted amines revealed a maximum adsorption capacity of ~124 mg g^−1^, which after functionalization increases according to 148 mg g^−1^ for C_KIT-6_–EDA, 158 mg g^−1^ for C_KIT-6_–DTA, 160 mg g^−1^ for C_KIT-6_–TTA and 181 mg g^−1^ for C_KIT-6_–TPA. This can be explained by the intensification of host–guest dynamic interplays occurring on the active sites with a high affinity for API species. Amine groups tend to change surface chemistry towards more basic. Additionally, covalently bonded nitrogen has an influence on the charge distribution and it increases the number of adsorption sites at the carbon surface via extra π–electrons [44,45].

**Figure 5:**
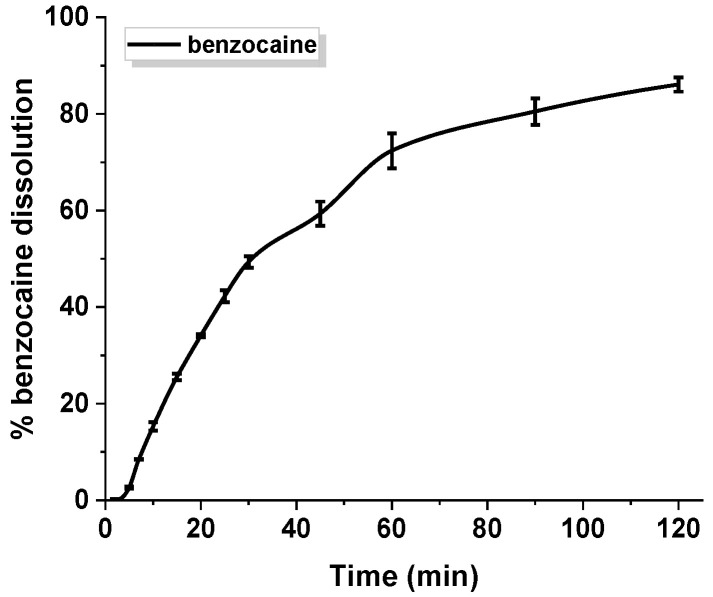
Dissolution profile of pure benzocaine. The standard deviation calculated from six measurements has been presented.

An in vitro release experiment of benzocaine was conducted in a buffer solution of pH 6.8 simulating oral cavity. Figure 5 depicts the profile of benzocaine dissolution contained in gelatin capsules. The study revealed that about 70% of pure benzocaine is present in the medium within 60 min of the process, and ca. 85% is present within 120 min. Drug release does not occur in an immediate way.

**Figure 6:**
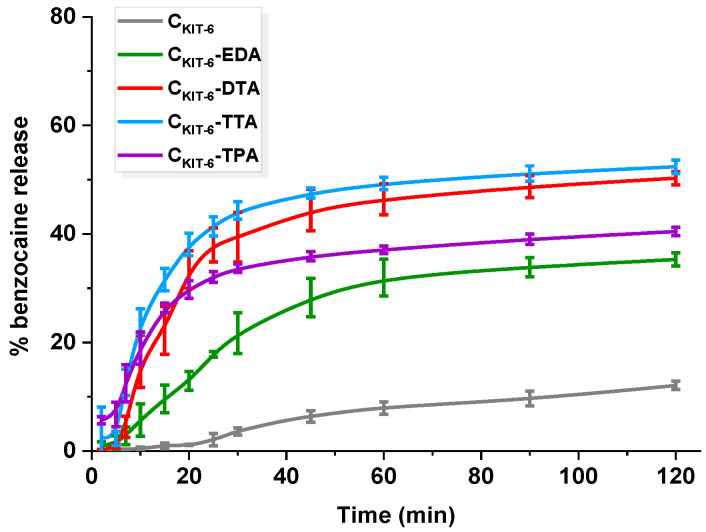
Release profiles of benzocaine from pristine and amine-grafted mesoporous carbon materials. The standard deviation calculated from six measurements for each material has been presented.

In the case of benzocaine-mesoporous carbon systems, the drug loading was estimated to be ~5 mg g^−1^. Despite carbons’ higher sorption capacities towards benzocaine, loading with the same amount of API provided a reasonable comparison of release behavior. All materials exhibited a high affinity towards benzocaine and significant liberation changes were observable in the first 120 min of the experiment depending on the chemical composition of the mesoporous carbons and their textural parameters (Figure 6). Pristine carbon revealed the smallest amount of benzocaine release reaching ~12%, which could be correlated with its most developed surface area and API–carrier interactions. The API molecules adsorbed in the pores of the carbon vehicle were slowly released into the receptor fluid due to their strong interaction with the pore walls. It should be mentioned that the interactions with pore walls play a significant role in the release from mesoporous matrices regardless of whether they are functionalized or not.

Notably, the presence of amine functional groups on the surface of carbons enhanced benzocaine dissolution. No significant changes in the quantities of benzocaine released, and process yield was observed for C_KIT-6_–DTA ~50% and C_KIT-6_–TTA ~52%. In the first hour, the rate of release differed for each material, e.g., in the case of C_KIT-6_–TTA, 40% of API was most rapidly released within 20 min, reaching 52% after 2 h. The C_KIT-6_–DTA sample behaved similarly, giving ~40% of drug released at the end of the process. A less rapid and rather controlled manner of benzocaine liberation exhibited C_KIT-6_–EDA (0.5% min^−1^), reaching 30% within 1 h, but within the second hour, only an additional 5% has been released, providing ~35%. Such improvement suggests different host–guest interactions and weaker attraction of benzocaine in the carbon. The release manner of benzocaine from C_KIT-6_–EDA is situated exactly between the behavior of pure mesoporous carbon and the carbons functionalized with amines of longer carbon chains. The surface of carriers modified with DTA, TTA, and TPA contains more amine groups that interact efficiently with benzocaine. Additionally, grafted functionalities may impede the access of API into the pores, in which adsorption is much stronger than on the external surface. Referring to the discussed release data, it transfers into faster benzocaine liberation from DTA-, TTA-, and TPA-functionalized carbons than from C_KIT-6_-EDA and pristine C_KIT-6_ samples. However, the smaller BET surface area and pore volume of functionalized carbons also have to be taken into account. Therefore, one may make an attempt stating that more benzocaine molecules in amine-grafted materials are present on the external surface, rather than within the pores. Thus, faster mass transfer is noticed, but still not complete. Quantitative comparison of the release of benzocaine from amine-functionalized OMCs with previous literature reports is difficult due to totally different experimental conditions being used. For instance, the amount of benzocaine diffused from emulsions such as ointment bases varied at the initial stage from 1 to 20%. The highest amount of API liberated within 2 h was 40 µg ml^−1^. However, tests were carried out via dialysis with the use of many additional reagents, e.g., HCl, NaNO_2_, NH_4_SO_3_NH_2_, BaCl_2_, and Ba(OH)_2_, whereas, in our case, only phosphate buffer was applied [46]. Other studies have been focused on reducing oral mucosal pain induced by orthodontic appliances using benzocaine-enriched wax. The wax was present in the mouth for 56 h and could be reapplied four times a day, which precludes comparison with our carbon carriers [47]. An attempt to compare release from carbon platforms was made with bi-layered tablets for local buccal administration. The conducted release of benzocaine from tablets was longer (2 days), but phosphate buffer utilized for studies had higher pH (7.4) [48]. Giving such examples we are only able to present mesoporous carbon carriers as new types of delivery systems, which, after appropriate modification, allow for controlling the processes of both the adsorption and release of benzocaine.

With the aim to establish a benzocaine diffusion mechanism from amine-functionalized porous carbons, the release data were fitted to five kinetic models (Table 2). Interestingly, there is no specific tendency towards one type of model, and API release behavior varies from each material. Non-modified sample C_KIT-6_ exhibited the highest R^2^ value to the Korsmeyer–Peppas model, with release exponent (n) being bigger than 0.89, associated with super case II transport [49]. In the case of using C_KIT-6_–EDA as a carrier for benzocaine, the zero-order model was the most accurate to describe the drug delivery process. It represents constant drug release, meaning similar doses were liberated per unit time. The last three carbon materials with the highest percentage drug release do not exhibit peculiarly high, near ~1 R^2^ for any model. However, calculations for API release from C_KIT-6_–DTA fitted it to the Hixson–Crowell model with R^2^ = 0.97, and from C_KIT-6_–TTA, and C_KIT-6_–TPA to the first-order model, with R^2^ = 0.96 and 0.97, respectively.

The two-value factors were calculated to confirm the similarities or differences in the benzocaine release profiles from mesoporous carbons. Their values (*f*_1_ > 15 and *f*_2_ < 50, Table 3) demonstrate that the benzocaine release profiles obtained for mesoporous carbon vehicles are different compared to the release profile of pure benzocaine.

**Figure 7:**
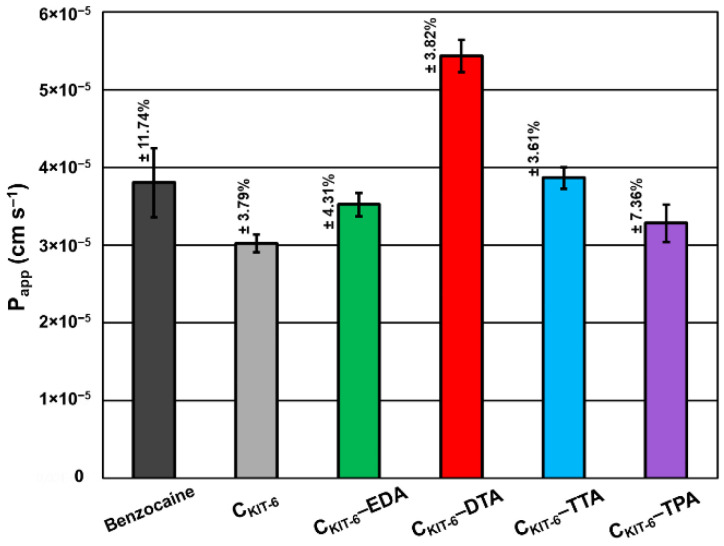
Apparent permeability coefficient (P_app_) of benzocaine from carbon carriers using the parallel artificial membrane permeability assay (PAMPA). The standard deviation calculated from three measurements for each material has been presented.

Based on in vitro permeability studies performed to simulate permeation through biological membranes in the gastrointestinal tract (Figure 7), the high permeability of benzocaine and its systems with mesoporous carbons was proved and expressed as *P_app_* values, which was higher than 1.0 × 10^‒6^ cm s^−1^. Pure benzocaine indicated 3.80 × 10^−5^ cm s^−1^ permeability, which has been slightly lowered by pristine C_KIT-6_ to 3.02 × 10^−5^ cm s^−1^ and materials modified with EDA (3.52 × 10^−5^ cm s^−1^) and TPA (3.28 × 10^−5^ cm s^−1^). C_KIT-6_ functionalization with TTA contributed to a small improvement of permeability with the following value: 3.86 × 10^−5^ cm s^−1^. However, the most significant increase in permeability was observed for the combination of benzocaine with C_KIT-6_–DTA giving 5.44 × 10^−5^ cm s^−1^.

## 4. Conclusions

The amine-grafted mesoporous carbons were successfully synthesized and applied as modern carriers for benzocaine—an active pharmaceutical ingredient whose effectiveness needs frequent dosing algorithms. It was shown that carbon carriers possessing the highest number of nitrogen-containing functional groups, stimulating the intensification of host–guest dynamic interplays with benzocaine, exhibited good adsorption capacities towards the drug. Benzocaine is linked to the surface of carbon materials mainly by hydrogen bonds, van der Waals forces, and electrostatic interactions. A high number of active sites for drug adsorption in the amine-functionalized samples causes a faster benzocaine delivery in a phosphate buffer solution of pH 6.8 simulating the oral cavity. The most controlled release was observed for carbon functionalized with ethylenediamine (C_KIT-6_–EDA) in the first 60 min, for which the zero-order model was the most accurate to describe the delivery process. It means that similar doses of benzocaine were liberated over unit time.

A clear limitation of the delivery platform developed is connected with using a pristine mesoporous carbon carrier containing only a small amount of surface functional groups. Interactions between benzocaine and pore walls of C_KIT-6_ sample are too strong to release a high percentage of the active ingredient after adsorption. Further studies will be focused on triethylenetetramine-functionalized C_KIT-6_, which released benzocaine in the highest amount (52%); therefore, it appears to be the most relevant vehicle.

The present work provides a rational basis for the design of the drug delivery profile, highlighting the role of different parameters, such as porosity, surface functional groups, and the stability of carriers. Through the optimization of host–guest interaction, it is possible to easily modulate the kinetics of the API release process.

## Figures and Tables

**Table 1:** Textural parameters of non-functionalized and amine-grafted porous carbons based on KIT-6 silica.

Material	BET Surface Area (m^2^ g^−1^)	Total Pore Volume (cm^3^ g^−1^)	Average Pore Diameter (nm)
C_KIT-6_	834	1.09	5.3
C_KIT-6_-EDA	507	0.62	5.6
C_KIT-6_-DTA	436	0.50	5.7
C_KIT-6_-TTA	359	0.39	5.9
C_KIT-6_-TPA	189	0.29	6.8

**Table 2:** Kinetics models applied to describe the release mechanism from pristine and amine-grafted mesoporous carbon vehicles.

Material	Zero Order		First Order		Higuchi		Hixson-Crowell		Korsmeyer-Peppas		
	k_0_ (h^−1^)	R^2^	k_1_ (h^−1^)	R^2^	k_H_ (h^−1/2^)	R^2^	k_HC_ (h^−1/3^)	R^2^	k_KP_ (h^−n^)	n	R^2^
**C_KIT-6_**	0.1117	0.9618	0.0012	0.9662	1.3930	0.9536	0.0018	0.9648	0.0081	1.7465	**0.9940**
**C_KIT-6-_EDA**	0.7750	**0.9931**	0.0035	0.9898	5.5285	0.9768	0.0130	0.9909	0.2870	1.2629	0.9709
**C_KIT-6_-DTA**	1.8220	0.9694	0.0103	0.9628	11.3668	0.9562	0.0326	**0.9747**	0.0116	3.0345	0.9680
**C_KIT-6_-TTA**	2.1878	0.9394	0.0120	**0.9574**	11.7347	0.9519	0.0396	0.9520	0.8392	1.2439	0.8594
**C_KIT-6_-TPA**	8.8455	0.9521	0.0079	**0.9678**	8.3871	0.9608	0.0262	0.9654	2.9144	0.7526	0.9373

**Table 3:** The two-factor (*f*_1_, *f*_2_) values for mesoporous carbons with benzocaine (B) systems, calculated and compared with pure benzocaine.

System	C_KIT-6_ + B	C_KIT-6_-EDA + B	C_KIT-6_-DTA + B	C_KIT-6_-TTA + B	C_KIT-6_-TPA + B
***f*_1_**	90.60	57.75	28.11	27.14	40.77
***f*_2_**	18.73	23.34	39.71	41.44	33.29

## Data Availability

Data available in a publicly accessible repository.
